# Pulmonary metastasectomy in pediatric patients

**DOI:** 10.1186/s12957-016-0788-6

**Published:** 2016-02-02

**Authors:** Basak Erginel, Feryal Gun Soysal, Erbug Keskin, Rejin Kebudi, Alaaddin Celik, Tansu Salman

**Affiliations:** 1 Istanbul Faculty of Medicine, Department of Pediatric Surgery, Istanbul University, Oguz Goker Caddesi, 5. Gazeteciler Sitesi, C-1 Blok No. 36, Akatlar Mahallesi, Besiktas, Istanbul, Turkey; 2 Department of Pediatric Hematology and Oncology, Istanbul University, Institute of Oncology, Istanbul, Turkey

**Keywords:** Pulmonary, Pediatric, Metastasectomy, Pulmonary metastasectomy, Pediatric surgery

## Abstract

**Background:**

This study aims to evaluate the outcomes of pulmonary metastasectomy resections in pediatric patients.

**Methods:**

We retrospectively reviewed the medical records of 43 children who were operated on in the Pediatric Surgery Clinic between January 1988 and 2014. Forty-three children (26 boys; 17 girls; mean age 10 ± 4.24 years, range 6 months–18 years) who underwent pulmonary metastasectomy resection were included in the study. The patients were evaluated based on age, gender, history of disease, surgical procedures, complications, duration of hospitalization, duration of chest tube placement, and procedure outcome.

**Results:**

Indications for pediatric resections were oncological. Metastasis was secondary to Wilms’ tumor in 14 patients, osteosarcoma in 7 patients, Ewing’s sarcoma in 5 patients, rhabdomyosarcoma in 5 patients, lymphoma in 3 patients, hepatoblastoma in 2 patients, and other tumors in 7 patients. A total of 59 thoracotomies were performed. Approaches utilized included unilateral posterolateral thoracotomy (*n* = 33), bilateral posterolateral thoracotomy (*n* = 8), and sternotomy (*n* = 2). Wedge resection was the procedure of choice (*n* = 44). In selected cases, 11 segmentectomies, 3 lobectomies, and 1 pneumonectomy were performed. There was no perioperative mortality. One patient suffered prolonged air leak and three patients from fever. All patients received chemotherapy. Radiotherapy was administered to 16 patients (37.2 %). Of those 16 patients, 7 had Wilms’ tumor, 6 had Ewing’s sarcoma/PNET, and 3 were rhabdomyosarcoma patients. During a median follow-up of 3 years, the overall survival was 74.4 %.

**Conclusions:**

Multidisciplinary treatment involving pediatric oncologists, surgeons, and radiation oncologists is necessary to obtain positive results in children who have pulmonary metastases of oncological diseases. Wedge resection is a suitable option for children because less lung tissue is resected.

## Background

Surgical excision is the gold standard in the pediatric metastases of pediatric age tumors. The loss of lung parenchyma and blood is important as it causes an increase in postoperative complications and morbidity [1]. Surgical excision is necessary to preserve the maximum amount of lung parenchyma but avoid local recurrence of disease. There is limited data in the literature concerning pulmonary metastasectomies in children. The purpose of our study is to evaluate our pediatric oncological patients who underwent pulmonary resections for metastases.

## Methods

Between 1988 and 2014, 43 children underwent surgery for pulmonary metastasectomy for oncological diseases at the Department of Pediatric Surgery. Indications of successful lung metastasectomy consisted of achieving control of the primary disease and observing the presence of sufficient lung capacity, as reported in the literature, in the patients [2, 3]. A pediatric hematology and oncology committee—including pediatric oncologists, pediatric surgeons, pediatric radiologists, and pediatric radiation oncologists—decides and assigns the indication for each lung metastasis.

The medical records of 43 children were retrospectively evaluated based on age, gender, history of disease, surgical procedure, complications, duration of hospitalization, duration of chest tube placement, and procedure outcome.

All statistical analyses were carried out with SPSS 22.0 (IBM, USA). Statistical significance was defined as *p* < 0.05.

## Results

Forty-three children were included in the study (26 boys; 17 girls), with a mean age of 10 ± 4.24 years (range 6 months–18 years). There was no statistically significant difference between the age and gender of the patients and overall survival rates (*p* = 0.029 and *p* = 0.48, respectively).

Thirty-two patients (74.4 %) had no symptoms. Fever (9.3 %) and fever with cough (9.3 %) were the next most common presentations. Chest radiography is performed in all patients. Thoracic computerized tomography (CT) was performed in all of the patients, and PET CT was performed in five patients.

All pediatric pulmonary resections were performed due to oncological diseases. The most frequent indication was pulmonary metastasis of Wilms’ tumor (32.5 %) in 14 patients. Other common causes of metastasis were osteosarcoma in 7 patients (16.2 %), Ewing’s sarcoma in 5 patients (11.6 %), rhabdomyosarcoma in 5 patients (11.6 %), lymphoma in 3 patients (6.9 %), and hepatoblastoma in 2 patients (4.6 %). Less common reasons were fibrosarcoma, PNET, neuroblastoma, teratoma, clear cell carcinoma, adrenal carcinoma, and testicular embryonal carcinoma, existing in one patient (2.3 %) (Table 1).

**Table 1:** Clinical features of the children

	Number	Percent
Gender		
Male	26	60.5
Female	17	39.5
Complaints		
None	32	69.7
Fever	4	9.3
Cough	3	6.9
Fever + cough	4	9.3
History of disease		
Wilms’	14	32.5
Osteosarcoma	7	16.2
Ewing	5	11.6
Rhabdomyosarcoma	5	11.6
Lymphoma	3	6.9
Hepatoblastoma	2	4.6
Fibrosarcoma	1	2.3
PNET	1	2.3
Neuroblastoma	1	2.3
Teratoma	1	2.3
Clear cell carcinoma	1	2.3
Adrenal carcinoma	1	2.3
Testis fibrosarcoma	1	2.3

**Fig. 1:**
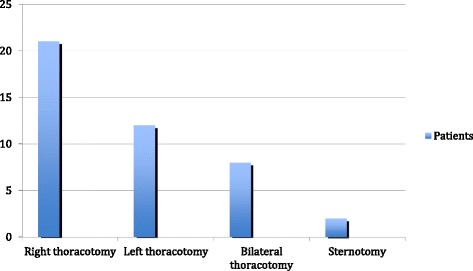
Our surgical approaches for pulmonary metastases

Left thoracotomy was performed in 21 patients, right thoracotomy was performed in 12 patients, bilateral thoracotomy was performed in 8 patients, and sternotomy was performed in 2 patients (Fig. 1).

**Fig. 2:**
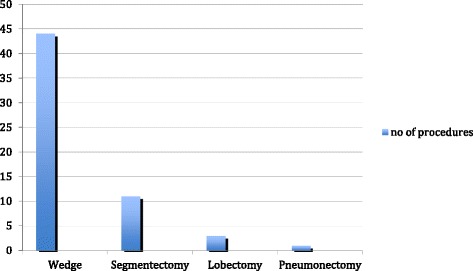
Surgical procedures performed

In terms of surgery, 44 wedge resections, 11 segmentectomies, 3 lobectomies, and 1 pneumonectomy were performed (Fig. 2). Wedge resections (74.5 %) were performed more frequently than anatomic resections (segmentectomy, lobectomy, pneumonectomy) (25.5 %).

Eleven (25.5 %) patients underwent more than one thoracotomy. Six of those underwent two thoracotomies (three with Wilms’ tumor, two with osteosarcoma, and one with Ewing’s sarcoma). Five of the 11 patients who received a rethoracotomy were operated on three times (three patients with osteosarcoma, two with Wilms’ tumor). In total, 59 thoracotomies were performed on 43 patients There was a statistically significant correlation between the number of recurrent thoracotomies in a patient and his/her survival (*p* = 0.005). It is likely to assume that patients in this group are in the advanced stages of the malignancy and thus they are receiving repeated thoracotomies.

The median number of resected nodules was 2 (1–17).

The mean length of hospitalization was 5.1 days. There was no perioperative mortality. We had no perioperative or postoperative complications, with the exception of fever in three patients and prolonged air leak in one patient.

A complete surgical resection with negative margins in the pathological reports was assessed in all of the patients.

After the early postoperative period, each patient was re-evaluated by the pediatric hematology and oncology committee to determine the appropriate post-operative adjuvant treatment. All patients received chemotherapy. Radiotherapy was administered to 16 patients (37.2 %). Of those 16 patients, seven had Wilms’ tumor, six had Ewing’s sarcoma/PNET, and three were rhabdomyosarcoma patients. During a median follow-up of 3 years, the overall survival was 74.4 %. Table 2 summarizes the percentage of survival among our 43 patients during a median follow-up period of 3 years (range 1–5 years). Survival analysis for each cancer type is given in Table 2. After pulmonary metastasectomy, the 3-year survival rate for Wilms’ tumor was 12/14 (85 %), osteosarcoma was 2/7 (28 %), Ewing’s sarcoma was 5/5 (100 %), rhabdomyosarcoma was 3/5 (60 %), lymphoma was 3/3 (100 %), and hepatoblastoma was 1/2 (50 %).

**Table 2:** Long-term (3-year) survival after pulmonary resection

	Number	Survivors
Wilms’	14	12/14 (85 %)
Osteosarcoma	7	2/7 (28 %)
Ewing	5	5/5 (100 %)
Rhabdomyosarcoma	5	3/5 (60 %)
Lymphoma	3	3/3 (100 %)
Hepatoblastoma	2	1/2 (50 %)
Fibrosarcoma	1	1/1 (100 %)
PNET	1	1/1 (100 %)
Neuroblastoma	1	1/1 (100 %)
Teratoma	1	1/1 (100 %)
Clear cell carcinoma	1	1/1 (100 %)
Adrenal carcinoma	1	0/1 (0 %)
Testis fibrosarcoma	1	1/1 (100 %)
Total	43	32/43 (74.4 %)

## Discussion

In children with solid tumors, the most common metastatic site is the lungs. Only a few decades ago, the survival of a child with cancer was very short when thoracic metastases were detected. However, with advances in CT technology, detection of even millimetric nodules is possible, and an improved understanding and recognition of the indications for surgical intervention has enabled these patients to survive much longer. In the literature, pulmonary metastasectomy is the part of standard treatment for adults with metastasis, but the data regarding surgical management of metastatic pulmonary nodules in children remains limited [4]. Success in prolonging patient survival rate via pulmonary metastasectomy is proven, particularly by retrospective studies involving osteosarcoma patients [5]. Incomplete resection is associated with short survival. In a series of studies by Tronc et al. evaluating 52 children with pulmonary nodules, four patients with incomplete resections died rapidly during the follow-up period [3]. In this study, pediatric oncology patients who underwent pulmonary resection for oncological metastatic nodules in our clinic are evaluated retrospectively. None of the resections involves primary pulmonary mediastinal mass.

Parida et al. evaluated 37 children with osteosarcoma and other cancers, for whom they performed thoracoscopic pulmonary metastasectomy after CT-guided needle hook replacement. They stated that thoracoscopic resection in preoperatively localized lung nodules is both a safe and efficient alternative to open surgery [6]. Han et al. evaluated 105 patients with pulmonary metastasectomy. They compared their thoracotomies (*n* = 46) and thoracoscopies (*n* = 62), which were undertaken to manage solitary lung metastases. They found that thoracoscopic metastasectomy is a promising option in small solitary pulmonary metastases [7]. One of the limitations of the study is the relatively small patient number, as is the study’s retrospective design. However, our morbidity and mortality are very low. One of the advantages of our open approach is that it permits palpation. CT scans sometimes underestimate the number of metastases [8]. The location of the metastatic nodule is also an important factor effecting predictive outcomes. Centrally located lesions may require anatomical resections such as lobectomy instead of the more common wedge resections performed for peripheral lesions. Letourneau et al. reported a very poor prognosis in patients with centrally located metastases in a retrospective evaluation of 115 osteosarcoma patients with pulmonary metastases [9].

Various prognostic factors are investigated to determine the effect of pulmonary metastasectomy on overall survival [10]. Optimal timing is also important in pulmonary metastasectomy as determined by Tanaka et al. A short interval between the detection of the tumor and pulmonary metastasectomy causes early relapses, according to their study [11].

We chose a second thoracotomy for our bilateral tumor cases rather than the bilateral simultaneous approach [12] utilized by Torre et al. In only two of our cases did we choose to perform a sternotomy incision and bilateral metastasectomy.

We prefer to perform lung sparing surgeries as much as possible, but it is also important to excise appropriate margins in order to avoid tumor recurrence. In most patients, a non-anatomic resection (wedge resection) was sufficient to adequately excise the tumor (44/59). Sublobar resections consisting of either segmentectomies or wedge resections are suitable surgical techniques to excise a pulmonary nodule, as they allow for the preservation of lung function in a growing child and avoid blood loss in smaller patients. We utilized sublobar resection in 55 of 59 (93.2 %) cases. As stated in the study performed by Döngel et al., sublobar resections are a suitable option for pediatric pulmonary metastasectomy [1].

There exists limited study data in the literature on long-term outcomes following pulmonary metastasectomy for removal of metastases secondary to solid tumors. Abel et al. evaluated the long-term survival of 20 children who underwent pulmonary metastasectomy. They suffered one early postoperative loss due to pneumonia, and four patients died later due to late metastases (mean of 29 months later). The overall mortality of their series was 25 % [12]. Hacker et al. evaluated 10 pulmonary metastasectomy patients, and during a mean follow-up period of 49 months, 8 patients (80 %) remained in complete remission [13]. Among our 43 pulmonary metastasectomy patients, there were 11 deaths, and our overall mortality was 11/43 (25.5 %), a percentage similar to the findings of their study.

Kayton et al. reported on their 50 years of cumulative experience performing metastasectomies in the pediatric population and established that pediatric pulmonary metastasectomy may be performed safely and effectively [14].

The present study consists of patients with many different histologies due to the rarity of many pediatric metastatic tumors. In the literature, most authors have evaluated pulmonary metastasectomies, which compromise various types of tumors [3, 13, 14, 15].

## Conclusions

The indications for pulmonary metastasectomy vary greatly in pediatric patients. Multidisciplinary treatment involving pediatric oncologists, surgeons, and radiation oncologists is necessary to obtain positive results in children who have pulmonary metastases of oncological diseases. Our retrospective study contributes to the increasing amount of data pertaining to both the indications for and outcomes of this procedure. Non-anatomic wedge resection is an ideal technique for pulmonary metastasectomy due to the preservation of lung parenchyma and decreased blood loss.
